# Efficacy and safety of aniseed powder for treating gastrointestinal symptoms of COVID-19: a randomized, placebo-controlled trial

**DOI:** 10.3389/fphar.2024.1331177

**Published:** 2024-01-16

**Authors:** Maryam Mosaffa-Jahromi, Hossein Molavi Vardanjani, Andrea Fuzimoto, Jennifer Hunter, Kamran Bagheri Lankarani, Mehdi Pasalar

**Affiliations:** ^1^ Research Center for Traditional Medicine and History of Medicine, Shiraz University of Medical Sciences, Shiraz, Iran; ^2^ Holistic Sync, Holistic Integrative Medicine, Rio, Brazil; ^3^ Health Research Group, Sydney, NSW, Australia; ^4^ Health Policy Research Center, Shiraz University of Medical Sciences, Shiraz, Iran

**Keywords:** COVID-19, gastrointestinal symptom, aniseed, abdominal pain, diarrhea, Persian medicine

## Abstract

**Background:** Gastrointestinal symptoms are prevalent amongst patients with a confirmed diagnosis of COVID-19 and may be associated with an increased risk of disease severity. This trial aimed to evaluate the efficacy and safety of aniseed (*Pimpinella anisum* L.) powder as an add-on therapy to standard care for treating gastrointestinal symptoms experienced by adults with an acute SARS-CoV-2 infection.

**Methods:** The study was a randomized parallel-group double-blinded placebo-controlled add-on therapy trial. Adults with an acute SARS-CoV-2 infection who did not require hospitalization and reported at least one gastrointestinal symptom in the preceding 48 h were assigned to either the aniseed or placebo group in a 1:4 ratio. All 225 participants (45 in the aniseed group and 180 in the placebo group) were instructed to use 25 g of powdered aniseed or placebo twice daily for 2 weeks. The primary outcomes were the proportion of patients who experienced an improvement of at least one point in the symptom score after adjusting for age group, gender, and time. Backwards stepwise logistic regression was applied to calculate the risk ratios. The clinical symptoms and adverse events were assessed at the beginning, 1 week later, and at the end of the trial (week two).

**Results:** Participants in the aniseed group were significantly more likely to report symptom improvement for abdominal pain [adjusted risk ratio (RR):0.55; 95% confidence interval (CI): 0.46–0.72], anorexia (RR:0.62; 95% CI: 0.47–0.82), and diarrhea (RR:0.19; 95% CI: 0.12–0.30), but not nausea/vomiting (RR:0.87; 95% CI: 0.71–1.08) or bloating (RR:0.87; 95% CI: 0.72–1.05). Two participants in the aniseed group and three participants in the placebo group reported mild to moderate adverse events.

**Conclusion:** This study showed that 2 weeks of aniseed powder containing trans-anethole (87%–94%) may help improve abdominal pain, anorexia, and diarrhea in COVID-19 patients. The findings align with the known biological, multitargeted activity of *P. anisum* and trans-anethole, which includes inhibiting SARS-CoV-2 along with other anti-infective, anti-inflammatory, antioxidant, hepatoprotective, and anti-dysbiosis properties. Multicenter trials with larger sample sizes and longer follow-up are warranted to confirm these findings.

**Clinical Trial Registration:** Iranian Registry of Clinical Trials (IRCT20120506009651N3).

## Introduction

Coronavirus disease 2019 (COVID-19) is caused by the severe acute respiratory syndrome coronavirus 2 (SARS-CoV-2). At the beginning of the COVID-19 pandemic, symptoms associated with acute respiratory tract infections, such as fever and cough, were the primary focus. As the pandemic has progressed and new variants have emerged, there is increasing awareness of the extrapulmonary manifestations of the disease. Over 70 different clinical manifestations have been identified, of which 10 are gastrointestinal (Luo et al., 2022).

The presence of gastrointestinal (GI) symptoms may precede pulmonary involvement (Jin et al., 2020; Poggiali et al., 2020). Anorexia (12.9%), diarrhea (8.1%), nausea (6.7%), constipation (5.5%), melena (5.3%), abdominal pain (3.7%) and heartburn (3.6%) are estimated to be the most common (Luo et al., 2022). While the overall prevalence is probably less than 20% and may not differ from the general population prevalence rates for neonates, children or pregnant women (Zeng et al., 2022), GI manifestations appear to be associated with an increased risk of COVID-19 severity, especially the presence of abdominal pain (Menon et al., 2021; Zeng et al., 2022). Serious complications, such as gastrointestinal bleeding and small intestine necrosis, have also been reported (Pan et al., 2020; Menon et al., 2021). However, it is currently unclear whether GI manifestations are associated with an increased risk of death, as the findings from systematic reviews are mixed (Menon et al., 2021; Wang et al., 2022).

Clinical guidelines for COVID-19 management provide non-specific recommendations for treating GI symptoms, such as antipyretics for pain and rehydration for vomiting or diarrhea (WHO, 2021). Persian medicine (PM) represents a rich source of potentially beneficial phytomedicines for various GI disorders (Avicenna, 2005; Aghili Shirazi, 2008). One example is the fruit of *Pimpinella anisum* L. (anise), also known as aniseed, which is dried and ground into an edible powder (*Sáfūf*). PM describes aniseed as possessing a warm and dry temperament that has analgesic, anti-bloating, anti-colic, carminative, and astringent properties (Ibn-al-Nafis, 2008; Aghili Shirazi, 2009).

There are over 150 species of the *Pimpinella* genus throughout the world, each containing different metabolites depending on geographical location. Trans-anethole (76.9%–93.7%) is often the main metabolite found in aniseed essential oil (Akbar, 2020).

Clinical trials that support PM traditional claims include a randomized, three-arm, double-blind, clinical trial, which reported that 4 weeks of treatment with an enteric-coated capsule of anise oil was more effective at eradicating the clinical symptoms of irritable bowel syndrome than both the placebo and the Colpermin^®^ (peppermint oil) intervention (Mosaffa-Jahromi et al., 2016). In another double-blind, randomized controlled trial, aniseed powder was more effective than a placebo (corn starch) powder at relieving postprandial distress syndrome symptom severity (Ghoshegir et al., 2015). Aniseed and its essential oil are common food products widely used across the globe and are generally considered to be safe. Reassuringly, neither of the aforementioned trails reported any serious adverse effects (Ghoshegir et al., 2015; Mosaffa-Jahromi et al., 2016).

Although mortalities from COVID-19 have dropped, there has been an increase in the number of people infected with the virus in different regions and at different times (Ma et al., 2021). Considering the global burden of the disease and the troubling GI manifestations that can affect the treatment process and the disease severity, finding effective methods to prevent and treat GI symptoms is important. The prevalence and potential risks of GI manifestations in COVID-19, the paucity of evidence-based clinical recommendations for managing these manifestations, and the traditional and emerging scientific evidence of aniseed as a safe and effective intervention for other non-specific GI conditions prompted us to conduct this study. This clinical trial aimed to evaluate the efficacy and safety of aniseed (*P. anisum* L.) powder for treating gastrointestinal symptoms experienced by adults with an acute SARS-CoV-2 infection.

## Methods

### Study design and protocol registration

This study is a randomized, parallel-group, double-blind, placebo-controlled, add-on therapy trial that was conducted from May to August 2022 at Motahari Clinic, affiliated with Shiraz University of Medical Sciences, Shiraz, Iran. Ethical clearance was provided by the Ethics Committee of Shiraz University of Medical Sciences (IR.SUMS.REC.1400.859) in accordance with the Declaration of Helsinki. The study protocol was registered on 2022-04-04 in the Iranian Registry of Clinical Trials (IRCT20120506009651N3).

Following registration and prior to recruitment protocol changes were made for pragmatic reasons. The intervention dose was increased from 5 g to 25 g per sachet. Symptoms were scored on a scale of 1 to 4 rather than with a frequency scale. The inclusion criteria were changed to exclude hospitalized patients, and patients only needed to have one, rather than all of the gastrointestinal symptoms.

### Primary and secondary outcomes

The efficacy outcomes were the proportion of patients who had an improvement of at least one point in the symptoms score including abdominal pain severity, anorexia severity, nausea and vomiting, bloating severity, and diarrhea frequency. Adverse events was the secondary outcome.

### Outcome measures

The primary endpoint for each efficacy outcome was the proportion of participants with a decrease of at least one point in their symptom score at the end of 2 weeks of treatment, adjusting for intervention, age group, gender, time, and body mass index (BMI). The secondary endpoint for each efficacy outcome was the change in weekly mean symptom score over the 2 weeks, adjusting for baseline score, gender, age and BMI. The secondary endpoints for adverse events were the number of participants who experienced one or more adverse events and the number and types of adverse events.

Symptom severity scores were assessed using the gastrointestinal questions from the Pars Farsi Questionnaire. The validity and reliability of this questionnaire were previously confirmed in the Pars Cohort Study (Gandomkar et al., 2017). The frequency of diarrhea per day was also recorded using a categorical question. Symptoms were scored on a four-point Likert scale (1 = none/mild and 4 = very severe), which participants recorded on a form at baseline, the end of week 1, and at the end of the study. Participants were instructed to report any side effects or unexpected symptoms in an open-ended question on the form and to contact the researchers by telephone. The participant questionnaire forms were collected at the end of the study.

### Intervention

Eligibility and enrollment of study participants were confirmed by internal medicine, gastroenterology, or infectious disease specialists at the Motahari Clinic. As an add-on therapy trial design, COVID-19 patients not requiring hospitalization were prescribed standard treatment according to the treatment protocols of the Iranian Ministry of Health (Rahmanzade et al., 2020). General recommendations regarding fluid intake, rest, nutrition, psychological support, and home oxygen therapy were provided to all patients. In addition, the participants were instructed to mix one 25 gr sachet of the intervention powder containing either the aniseed or a placebo, in 250 mL of water to be administered twice daily, 1 h after breakfast (9 a.m.) and lunch (3 p.m.), for 2 weeks.

### Formulation of aniseed powder

The Pharmaceutics Laboratory of Shiraz School of Pharmacy was responsible for preparing the formulations. An oral powder formulation of aniseed was selected to facilitate optimal delivery to the GI system (Avicenna, 2005). Prior to grinding with an ultra-centrifugal electric mill, contaminating particles (soil, dust, etc.) were removed manually from the aniseeds. The aniseeds were then powdered and sieved using mesh size number 50. The obtained powder was sealed in polyethylene plastic bags and kept at 10°C until the quality control analysis at Shiraz University of Medical Sciences, school of pharmacy. After the quality was confirmed, the aniseed powder was mixed with rock candy powder (Sepahan Industrial Co., Iran) in a ratio of 2:1 and then sieved again using mesh size number 50. After assessing the particle size, pH, volumetric mass, moisture content, ash percentage, flow, and organoleptic characteristics, the product was packaged in sachets with a medicinal net weight of 25 gr (±5 gr), followed by a product weight confirmation test.

### Formulation of placebo powder

To prepare the placebo powder, white wheat flour (Zar Macaron Industrial Co., Iran) and rock candy powder (Sepahan Industrial Co., Iran) were mixed in the same ratio mentioned above and packed in 25 g sachets. The drug and placebo were matched for packaging, color, and appearance, but not taste.

### Identification and cultivation of anise plant

Aniseed is the fruit of *P. anisum* L., which is native to Iran. Samples of the fruit were purchased from three different sources (a local market in Shiraz city, a traditional medicine shop in Shiraz bazar, and a medicinal plant garden in School of Pharmacy, Shiraz); each sample was collected in Shiraz, Fars province, Iran [latitude and longitude coordinates are: 29.591768, 52.583698.] in summer 2021, and a herbarium of the entire plant was prepared. The plant was identified and confirmed by a plant species specialist (herbarium number: TP-772).

### Standardization and quality control of aniseed powder

In addition to noting the appearance properties, the aniseed powder metabolites were identified and quantified based on the U.S., British, and European pharmacopeias. The anise essential oil in the powder was standardized using different methods, focusing on its main metabolite, trans-anethole (87%–94%) at the School of Pharmacy, Shiraz University of Medical Sciences. Trans-anethole is a key distinguishing metabolite found in aniseed (Ghosh et al., 2019).

### Thin-layer chromatography analysis of anise essential oil and trans-anethole

The anise essential oil underwent thin-layer chromatography (TLC) using a mixture of ethyl acetate and methanol in a ratio of 19:1. Subsequently, the anise essential oil and trans-anethole were spotted on a TLC plate, both diluted in 5 mL dichloromethane, and the plates were dried. The resulting spots were visualized by spraying the trans-anethole detector on the samples. Afterwards, the plates were placed on a hot plate, causing the two corresponding spots to undergo derivatization with similar retention factors, indicating the similarity between the two compounds.

### Gas chromatography analysis of anise essential oil and trans-anethole

The anise essential oil and pure trans-anethole were analyzed using a gas chromatography/flame ionization detector (GC/FID) apparatus. The analysis was conducted on a BRUKER gas chromatograph equipped with a 5CB capillary column and connected to an FID. Nitrogen was used as the carrier gas at a flow rate of 1 mL/min, and the split ratio was set at 1:100. The injector temperature was maintained at 250°C, while the detector temperature was set to 280°C. The column temperature was programmed to increase linearly from 60°C to 210°C at a rate of 5°C/min and then held at 210°C for 15 min. The retention time for both the anise essential oil and trans-anethole exhibited identical values.

Additionally, the essential oil profile of an anise oil sample was determined by utilizing chromatography/mass spectrometry (GC/MS). The analysis was performed using an Agilent 6,850 Series II instrument equipped with a fused silica HP-5 capillary column (30 m × 0.25 mm, 0.25 μm film thickness) and an Agilent Mass Selective Detector MSD 5973. The ionization voltage was set at 70 eV, the electron multiplier energy at 2,000 V, and the source temperature at 250°C. Mass spectra were scanned within the range of 35–450 amu at a scan time of 5 scans/s. The transfer line temperature was maintained at 295°C.

The identified components were established by comparing their mass spectra with those available in the Willey and Adams libraries. Additionally, the Kovats retention index and information reported in the literature were taken into consideration. Among the various components identified through GC/MS analysis, trans-anethole was specifically determined as the major compound in the essential oil of this collected sample.

### Microbial limits testing

Microbial limits tests were conducted to determine the presence of microorganisms in the sample. These tests included counts of Gram-negative bacteria, *Escherichia coli* and *Salmonella* to assess the microbiological safety and quality of the sample. By performing these tests, it was evident that the overall microbial contamination was within the normal range, ensuring that the sample met the required standards for purity and safety.

### Inclusion criteria

Included were patients who met the following criteria: 1) acute COVID-19 infections confirmed by a positive PCR test; 2) having at least one gastrointestinal symptom (e.g., nausea, vomiting, abdominal pain, diarrhea, anorexia, constipation) within 48 h before the examination; 3) mild to moderate lung involvement (less than 30% according to chest computed tomography scan) without the need for immediate hospitalization; 4) between 18 and 70 years of age; 5) available for subsequent evaluations during the study; 6) voluntary participation after signing the informed consent form.

### Exclusion criteria

Excluded were patients with a history of 1) chronic gastrointestinal diseases (e.g., gastrointestinal reflux, hiatus hernia, peptic ulcer, irritable bowel syndrome, inflammatory bowel disease); 2) allergy to any kind of botanical drug product; 3) serious or critical conditions, such as congestive heart failure, kidney failure, or liver failure; 4) diabetes, thyroid disease, high blood pressure, or any other contraindications to the use of botanical drug products as determined by the researchers; 5) the presence of any venous thrombosis risk factors; 6) pregnant or breastfeeding women; 7) simultaneous participation in another clinical trial; 8) increasing severity of disease or need for hospitalization during the trial; or 9) use of treatments outside of the standard protocol for the treatment of COVID-19.

### Randomization and allocation concealment

Restricted randomization, using random permuted blocks of five, with an allocation ratio of 1:4 was applied. A free online *Saghaei* random allocation software was used to generate a random sequence (https://mahmoodsaghaei.tripod.com/Softwares/randalloc.html). Each generated random sequence was recorded on a card by a research assistant who had no other role in the study. The card was then sealed within an envelope, and a three-digit serial number was recorded on the outer surface of each envelope. The envelopes were opened sequentially according to the order of participant enrollment, and participants were assigned to either the aniseed or placebo group. Another research assistant, was blinded to the group allocation, dispensed the interventions.

### Blinding

To maintain blinding, participants, physicians, and personnel involved in executing the study procedure were unaware of the group allocation. Participants, physicians, and personnel involved in executing the study procedure were blinded to the group allocation. Blinding of study personnel, including those involved in data entry and preparing the data for analysis, was maintained until the database was locked. The codes were then opened by the supervisor of the research team.

### Sample size

The sample size calculations were based on an anticipated prevalence of diarrhea in patients with COVID-19 of 49.5% (Fang et al., 2020), with 80% statistical power, 5% type 1 error, and a 50% greater impact for alleviating GI symptoms. Due to financial limitations, the ratio of participants in the experimental and control groups was set at 1:4. Initially, the sample size for the experimental group was calculated at 40 people, and then extended to 45, considering a 15% probability of dropout during the study. Hence, out of a total of 225 eligible patients, 45 were recruited in the experimental group, and 180 were recruited in the control group.

### Statistical analysis

An intention to treat analysis was conducted. When comparing differences in baseline characteristics between the two groups, the independent *t*-test was used for continuous variables, and the chi-squared test was used for categorical variables. For multivariate analysis of the risk ratio and repeated measures analysis of change in mean symptom score over the 2 weeks, generalized estimating equation analysis was employed. The backward elimination technique with an elimination criterion of a *p*-value >0.3 was used to optimize the model. The following covariates were included in the full saturated model: intervention, age group, gender, time, and BMI. Stata software (version 11.2, Texas, United States) was used for statistical analysis. A *p*-value <0.05 was considered significant.

## Results

**FIGURE 1:**
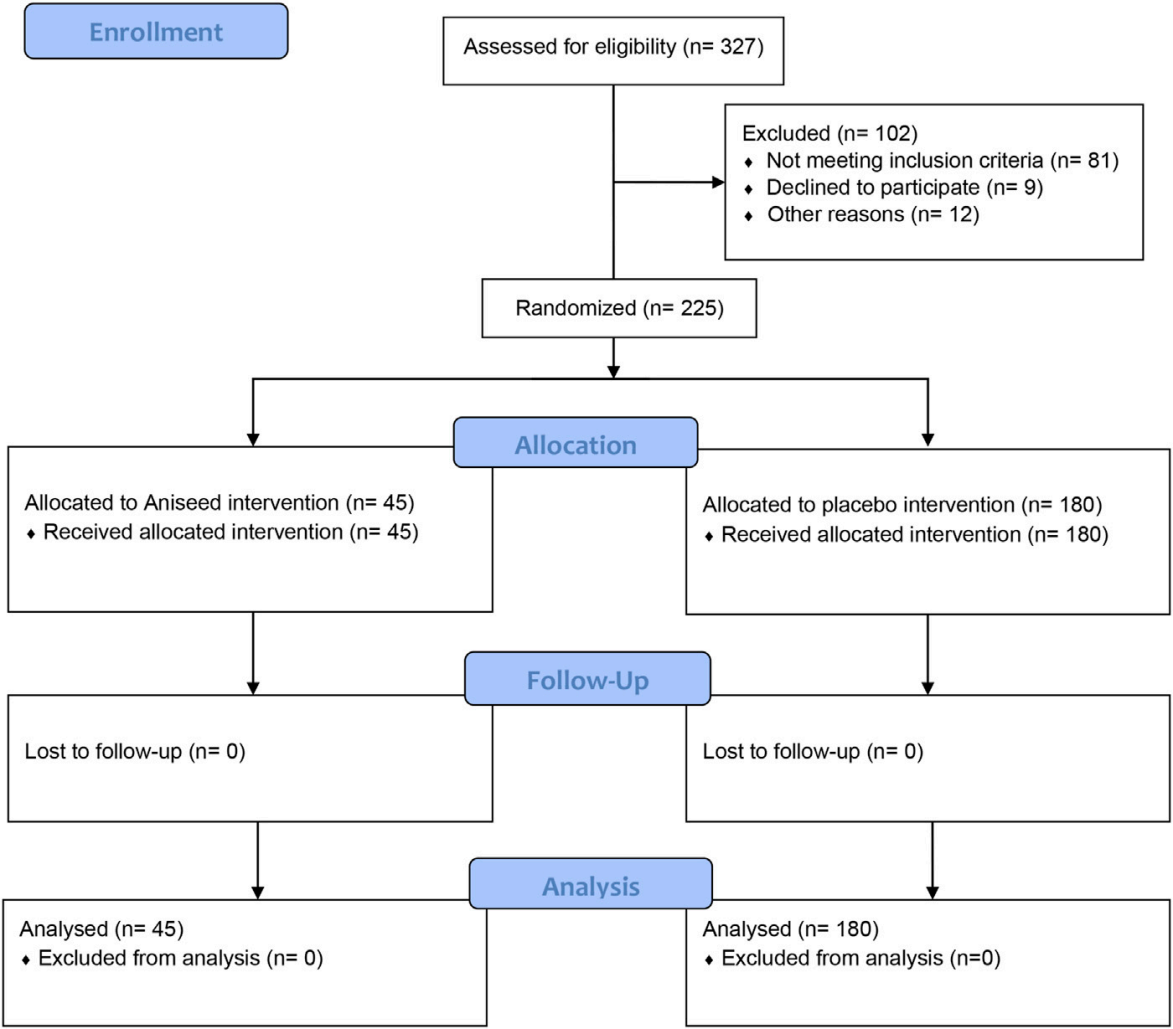
CONSORT flowchart of participants.

Out of 327 adults who were assessed for eligibility, 225 were randomized into the placebo (n = 180) or aniseed (n = 45) intervention group (Figure 1). All participants received the allocated intervention, and no participants were lost to follow-up or excluded from the analysis. We had no missing outcome data for the 15 symptom items (i.e., 5 questions at baseline, week 1 and week 2).

The average age of the participants was 40.96 years (SD 10.70) in the aniseed group and 44.63 years (SD 13.68) in the placebo group (*p* = 0.094). Sixty percent of the participants were female. Although there were no significant differences in baseline mean anorexia severity (*p* = 0.150), abdominal pain severity (*p* = 0.075), or diarrhea frequency (*p* = 0.445) scores, both the mean nausea and vomiting severity score (*p* = 0.002) and mean bloating severity score (*p* < 0.001) were significantly lower in placebo group. The participant characteristics are summarized in Table 1.

**TABLE 1:** Characteristics of participants.

Variable	Placebo group (n = 180)	Aniseed group (n = 45)	*p*-value
Mean age (years)	44.63 (SD 13.68)	40.96 (SD 10.70)	0.094
Less than 20	7 (3.8%)	1 (2.2%)	0.693
20 to 39	73 (40.6%)	21 (46.6%)	
≥40	100 (55.6%)	23 (51.2%)	
Gender			
Male	113 (62.7%)	23 (51.2%)	0.152
Female	67 (37.2%)	22 (48.8%)	
Ethnicity			
Fars	170 (94.4%)	42 (93.3%)	0.775
Other	10 (5.6%)	3 (6.7%)	
Education			
Diploma and Less than diploma	84 (46.6%)	15 (33.4%)	0.107
More than diploma	96 (53.4%)	30 (66.6%)	
Marital status			
Single	60 (33.3%)	17 (37.7%)	0.574
Married	120 (66.7%)	28 (62.3%)	
Smoker			
No	125 (69.4%)	35 (77.7%)	0.270
Yes	55 (30.6%)	10 (22.3%)	
Mean BMI			
<30	154 (85.6%)	40 (88.9%)	0.562
≥30	26 (14.4%)	5 (11.1%)	
Gastrointestinal symptoms			
Anorexia severity score	2.06 (SD 0.98)	2.29 (SD 0.82)	0.150
Nausea & Vomiting severity score	2.07 (SD 0.98)	2.58 (SD 1.01)	0.002
Abdominal pain severity score	2.75 (SD 0.90)	3.0 (SD 0.67)	0.075
Bloating severity score	1.80 (SD 0.87)	2.54 (SD 1.01)	<0.001
Diarrhea frequency score	5.62 (SD 1.77)	5.83 (SD 1.11)	0.445

Abbreviation: BMI, Body mass index; SD, Standard deviation.

After adjusting for age group, gender, BMI, and time, participants in the aniseed group were significantly more likely to report symptom improvements in abdominal pain (risk ratio (RR): 0.55; 95% confidence interval (CI): 0.46, 0.72), anorexia (RR: 0.62; 95% CI: 0.47, 0.82), and diarrhea (RR: 0.19; 95% CI: 0.12, 0.30) in patients with COVID-19 but, not significant effect on nausea/vomiting or bloating (Table 2).

**TABLE 2:** Effect of aniseed product on risk of reducing gastrointestinal symptoms of COVID-19 patients.

Gastrointestinal symptom as outcome	Unadjusted RR to compare aniseed vs. placebo (95% CI)	Adjusted RR to compare aniseed vs. placebo (95% CI)	*p*-value
Abdominal Pain	0.57 (0.47, 0.76)	0.55 (0.46, 0.72)	<0.001
Anorexia	0.64 (0.51, 0.84)	0.62 (0.47, 0.82)	0.001
Nausea/vomiting	0.88 (0.72, 1.07)	0.87 (0.71, 1.08)	0.268
Diarrhea	0.21 (0.13, 0.30)	0.19 (0.12, 0.30)	<0.001
Bloating	0.84 (0.64, 1.02)	0.87 (0.72, 1.05)	0.153

Abbreviations: RR, risk ratio; CI, confidence interval.

*p-value is not statistically significant at 0.05.

**FIGURE 2:**
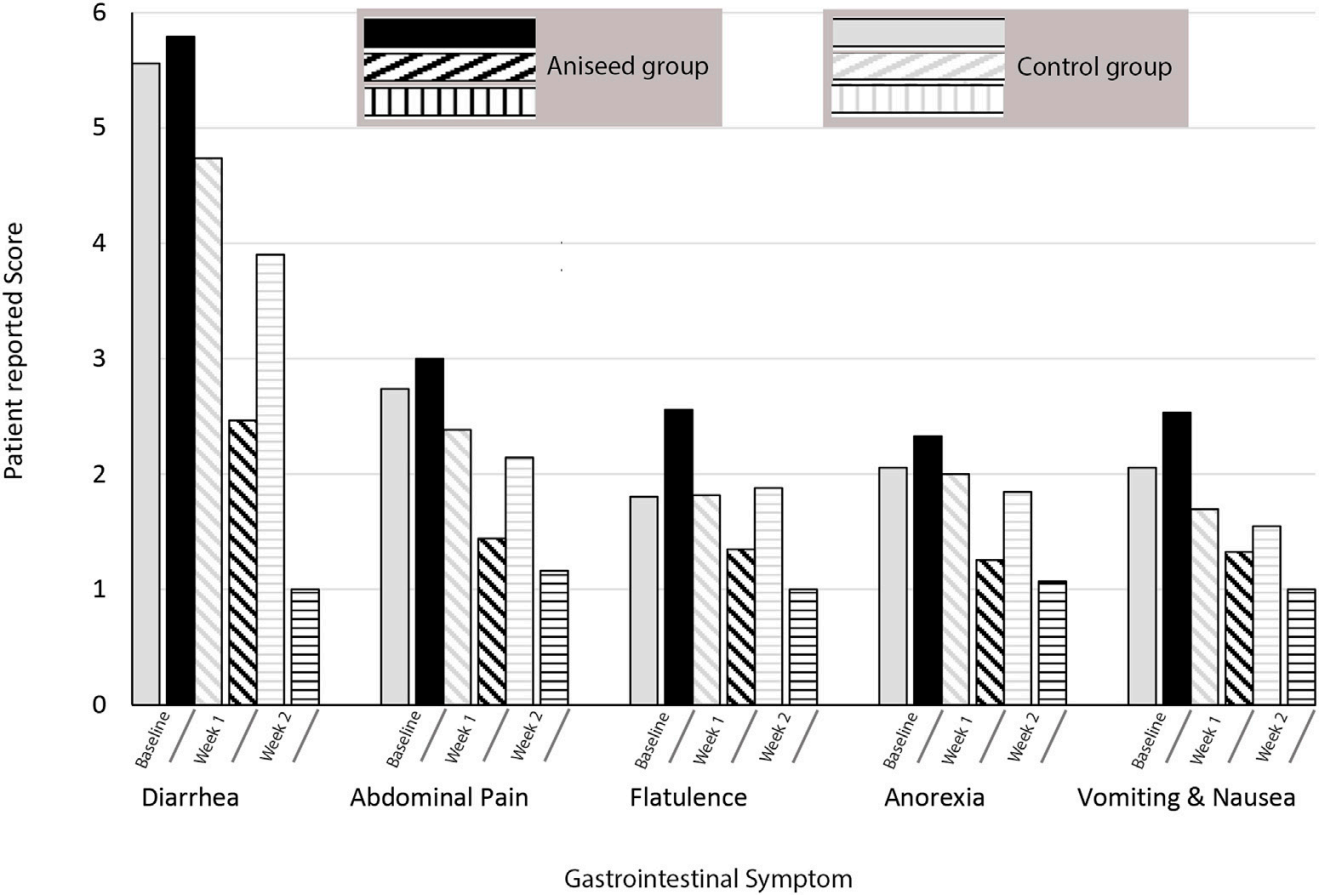
Mean scores of gastrointestinal symptoms at the baseline, week 1, and week 2.

The mean scores of GI symptoms at the beginning, end of week one, and end of the study (week two) are shown in Figure 2. After adjusting for mean baseline score, age group, gender, BMI and the change in weekly mean symptom score over the 2 weeks, participants in the aniseed group were significantly more likely to report symptom improvements in mean scores for abdominal pain (*p* < 0.001), anorexia (*p* = 0.001), diarrhea (*p* < 0.001).

No serious adverse events were reported. In the aniseed group, two participants complained of mild throat itching, and one of mild thirst. In the placebo group, three participants complained of a mild cough, and two of moderate abdominal cramp. None of these participants stopped the intervention nor withdrew from the study.

## Discussion

This study is novel in two aspects. Firstly, it investigated the effects of botanical drug on gastrointestinal symptoms experienced by patients with COVID-19 using a double-blinded, placebo-controlled add-on therapy trial study design. Secondly, it explored the efficacy of a popular PM botanical drug, aniseed (*P. anisum* L.), that has received little attention in the context of viral infections. Our results showed that daily consumption of the aniseed formulation significantly reduced three out of the five GI symptoms–abdominal pain, anorexia, and diarrhea. Furthermore, the formula was well tolerated and no dangerous or life-threatening adverse events were observed.

Although the current study is significant for the introduction of a new botanical drug metabolite to alleviate some common gastrointestinal symptoms in COVID-19 patients, it had certain limitations such as not recording the patients’ diet nor difference in taste of aniseed and placebo that may have impacted blinding, absence of molecule docking results, and lack of a long-term follow-up for the patients. Additionally, no statistical adjustments for multiple comparison were made, however, a Type 1 error is unlikely as the *p*-value was very small for all three significant differences in the primary endpoints.

Notwithstanding these limitations, given these positive findings, we explored the potential mechanisms by which *P. anisum* and some of its metabolites, especially trans-anethole, could have improved symptoms by suppressing the SARS-CoV-2 and/or intervening in the pathophysiological process of COVID-19.

### Inhibition of SARS-CoV-2 proteins by aniseed–*in silico* studies

Some *in silico* studies showed the potential of *P. anisum* to inhibit the SARS-CoV-2 Spike (S) protein and the Chymotrypsin-like protease or main protease (3CL^PRO^ or M^PRO^). The receptor binding domain (RBD) of SARS-CoV-2 S glycoprotein binds to angiotensin-converting enzyme 2 (ACE-2) receptors of host cells to gain entry and replicate. ACE-2 receptors are abundantly expressed in human tissues, not only in the respiratory system (nasopharynx and bronchus), heart, kidneys, and male reproductive system, but also in the enterocytes of the small intestine, colon, and rectum, and gallbladder, and pancreas (Pontén et al., 2008). The expression of ACE-2 mRNA and protein in the gastrointestinal tract is 100 times higher than in the lungs (Hikmet et al., 2020). While the S protein allows attachment and viral entry into target cells, the 3CL^PRO^ is one of the proteins that cleave large polyproteins, and release replicative proteins that allow the process of viral transcription, translation, and replication. Drugs that can block the S protein and the 3CL^PRO^ are considered good drug candidates to fight viral infection. Thus, the gastrointestinal tract is a credible doorway for SARS-CoV-2 access.

Thus, these *in silico* studies show potential mechanisms by which *P. anisum* and its phytochemicals could directly inhibit SARS-CoV-2 replication and reduce COVID-19 gastrointestinal symptoms.

### Antiviral properties of aniseed—*in vitro* studies

Although *in vitro* studies on the antiviral action of *P. anisum* and trans-anethole against RNA viruses are scarce, the water extract of *P. anisum* has demonstrated antiviral activity against herpes simplex (HSV-1 and HSV-2), cytomegalovirus (HCMV), and measles virus (Lee et al., 2011). In an *in vitro* study, trans-anethole (from star anise essential oil or *Illicium verum*) was one of the 6 tested metabolites that suppressed the HSV-1 infectivity by > 90% at a maximum noncytotoxic concentration. In this experiment, the herpes virus was incubated for 1 h with various concentrations of botanical drug metabolites before host infection. The authors concluded that the successful inhibiting metabolites directly inactivated the virus probably interfering with virion envelope structures or blocking structures that are necessary for cell entry (Astani et al., 2011).

### Anti-inflammatory properties of aniseed

The invasion of SARS-CoV-2 into cells of the GI tract causes breakage of the mucosal barrier and promote the production of inflammatory factors (Yi et al., 2021). Studies have shown abnormal levels of cytokines and chemokines in patients with COVID-19 such as IL-1, IL-2, IL-4, IL-6, IL-7, IL-10, IL-12, IL-13, IL-17, M-CSF, G-CSF, GM-CSF, IP-10, IFN-γ, MCP-1, MIP 1-α, hepatocyte growth factor (HGF), TNF-α, and vascular endothelial growth factor (VEGF) (Costela-Ruiz et al., 2020). However, the cytokine and chemokine profile may vary depending on the COVID-19 14-day disease phase, disease severity, location where the infection started (respiratory or digestive system), and the section of the infected GI tract (Jiao et al., 2021). Natural agents that can modulate inflammation in the GI tract during the SARS-CoV-2 infection would likely contribute to symptom improvement.

The chemical structure of the *P. anisum* plant was previously investigated and reported in detail. The presence of trans-anethole as a main metabolite enables us to identify possible mechanisms to explain the beneficial effects of the aniseed formulation. In Yanarlia et al.'s study, *P. anisum* and its volatile essential oil metabolites demonstrated anti-inflammatory activity, reducing the expression level of pro-inflammatory cytokines IL-1 and IL-8 in the epithelial cells of the trachea and primary bronchi in an animal model of lipopolysaccharide-induced lung inflammation (Iannarelli et al., 2018). Another controlled study found that aqueous extract of aniseed significantly reduced the gene expression of IL-5 and IL-13 and the protein levels of inflammatory cytokines in the lung tissue of laboratory mice with allergic asthma treated (Dargahi et al., 2021).

Anti-inflammatory and gastroprotective effects of trans-anethole have also been demonstrated in experimental model via different mechanisms (Yi et al., 2021; da Rocha et al., 2023; Yu et al., 2022; Tong et al., 2023). These anti-inflammatory effects are similar in strength to those of indomethacin (Tas et al., 2006) and may also help explain the mechanism behind relieving gastrointestinal symptoms. For example, abdominal pain, as a probable predictor of severe COVID-19 (Hayashi et al., 2021), can be caused by the stimulation of sensory nerves (with an increase in cytokines and chemical inflammatory mediators) (Coates et al., 2013), and the anti-inflammatory effect of the *P. anisum* essential oil may impede this pathway. It has also been shown that myalgia caused by COVID-19 has an inflammatory basis (Hasan et al., 2021), and the anti-inflammatory effect of trans-anethole is also worth considering in this context. In addition, the analgesic effects of anise’s essential oil have been shown to be comparable to those of drugs like aspirin and morphine (Tas et al., 2006).

A recent cross-sectional study involving older adults identified inflammation to be as a major independent risk factor for reduced appetite (Sieske et al., 2019). Therefore, the anti-inflammatory metabolites of *P. anisum* may help explain the improvements in anorexia in our study. Diarrhea, another common gastrointestinal symptom in patients with COVID-19, was reduced significantly with the use of the aniseed formulation in the current study. This symptom may originate from inflammation of the intestinal cells (Sweetser, 2012), which would explain the therapeutic effect of the formulation, considering its anti-inflammatory properties. On the other hand, the lack of affecting bloating in this study may be due to the fact that compounds containing anti-inflammatory substances can cause or aggravate bloating (Suzuki et al., 2007). While strong glucocorticoids like dexamethasone offer mild anti-nausea/vomiting effects due to their anti-inflammatory function (Chu et al., 2014), *P. anisum* and its active metabolute (trans-anethole) are not among the strong anti-inflammatory substances compared to these metabolites. This may explain the observed ineffectiveness of the aniseed formulation in treating nausea/vomiting.

### Hepatoprotective and antioxidant effects of aniseed

The COVID-19 infection can lead to liver damage, abnormal liver function, and elevated liver enzymes, with gastrointestinal and abdominal clinical manifestations (Ma et al., 2020). COVID-19-associated liver injury has been confirmed through autopsy and biopsy and the main findings are hepatic steatosis, mild lobular and portal inflammation, congestion/sinusoidal dilation, lobular necrosis, and cholestasis (Yang et al., 2023). COVID-19-related liver injury may be due to drug-induced liver injury, systemic inflammation, and hypoxia-ischemia reperfusion, but liver damage from a direct SARS-CoV-2 attack is uncertain (Zhong et al., 2020). Also, recent studies suggest that excessive production of reactive oxygen and nitrogen species (RONSs) and an unbalanced antioxidant-oxidant system play an important role in COVID-19 disease process (Suhail et al., 2020). Since ACE-2 receptors contribute to lowering oxidative stress which in turn modulates the binding affinity of both SARS-CoV-2 S protein and ACE-2, the authors concluded that, therapeutics that reduce oxidative stress could prevent viral binding to host cells and have a beneficial effect in the early stage of viral infection (Suhail et al., 2020). Moreover, it has been suggested that glutathione deficiency may be implicated in the pathogenesis of severe COVID-19 (Polonikov, 2020).

In a study investigating the effects of trans-anethole in hepatotoxicity associated with high doses of acetaminophen, this compound prevented the upregulation of proinflammatory mediators involved in the hepatotoxicity such as nitric oxide (NO), TNF, IL- α, MIP-1 α, and MCP-1 (da Rocha et al., 2017). In a murine research model, trans-anethole protected the liver against diabetes-induced hepatic injury by reducing blood glucose levels, liver enzyme activity, food and water intake, the intensity of weight loss, serum triglycerides, total cholesterol, low density lipoprotein, and increased high density lipoprotein (Samadi-Noshahr et al., 2021).

Also, in an *in vitro* and *in vivo* study, the researchers verified the hepatoprotective effects of n-hexane extracted from *P. anisum* against carbon tetrachloride-induced hepatotoxicity. N-hexane lowered serum transaminases levels, lactate dehydrogenase activity, liver histological changes, and cells death (*in vitro* and *in vivo*), and increased glutathione (GSH) and decreased thiobarbituric acid-reactive (*in vitro*) (Jamshidzadeh et al., 2015). The authors concluded that n-hexane extract of anise has hepatoprotective activity through its antioxidant metabolites. In fact, the water and ethanol extract of *P. anisum* seed exhibited antioxidant activity in all concentrations tested. Both extracts had strong free radicals, superoxide anion, hydrogen peroxide scavenging, and metal chelating activities when compared to control substances (Gülçın et al., 2003).

### Action of aniseed on COVID-19 microbiome and gut epithelial barrier

Besides GI mucosal damage, the gastrointestinal microbiome can be greatly affected by the SARS-CoV-2 infection. When comparing the microbiome composition of COVID-19 patients and uninfected controls, the COVID-19 patients had a depletion of beneficial microorganisms that help maintain GI immune and barrier functions (Sun et al., 2022). There was also an increase in pathogenic species with significantly higher quantities in severe patients that tended to decrease when patients improved. The intestinal dysbiosis caused altered immune responses, gut barrier dysfunction, and an increase in circulating levels of lipopolysaccharide-binding protein in severe patients. Increased permeability of the intestinal epithelial lining allows viral translocation into the bloodstream and potential systemic dissemination (Sun et al., 2022). Also, shotgun metagenomic sequencing analyses detected an enrichment of opportunistic pathogens and depletion of beneficial commensals organisms in hospitalized COVID-19 patients which correlated with disease severity (Zuo et al., 2020). Interventions that can restore microbiome balance, protect epithelial lining, and modulate immune responses would likely help in this scenario.

Essential oil extracted from *P. impinella* species has been found to counteract both Gram-negative and Gram-positive bacteria (Nasır and Yabalak, 2021). In an animal study, anethole opposed *E. coli* K88, enriched the abundance of beneficial flora, protected intestinal barrier function, upregulated mucosal layer and tight junction proteins, reduced plasma concentrations of IL-1β and TNF-α (*p* < 0.1), and decreased the mRNA expression of TLR5, TLR9, MyD88, IL-1β, TNF-α, IL-6, and IL-10 (Yi et al., 2021). Also, the star anise (*I. verum)* essential oil, which also may contain a fair amount of trans-anethole was effective at killing beneficial and pathogenic organisms of the GI flora, although it was less active toward *Lactobacilli* than *Bifidobacteria* or potentially pathogenic organisms (Hawrelak et al., 2009). Thus, anethole-containing essential oils may have a modulatory effect on the gut microbiome and could assist in COVID-19 pathological process. Since diarrhea is a common symptom of COVID-19 and this symptom can also be attributed to intestinal dysbiosis, the improvement seen in our study could be partly explained by a beneficial action of *P. anisum* and its metabolites on the patients’ gut microbiome and intestinal epithelium barrier.

### The potential effects of aniseed on the gut-lung axis in COVID-19

In a novel non-human primate model research, two types of SARS-CoV-2 inoculations were tested, intranasal and intragastric inoculations (Jiao et al., 2021). Interestingly, both separate inoculations caused infection, pathological changes, and viral shedding in the respiratory and digestive tissues (pneumonia and GI dysfunctions), induction of inflammatory cytokines, and gut barrier impairment. The intragastric inoculation induced a larger amount of inflammatory cytokines which may then spill over to the respiratory tissues via the bloodstream and lymphatic system. The authors concluded that GI inflammatory factors are a potential bridge for viral pathogenesis between the respiratory and GI systems (Astani et al., 2011). The gut-lung axis or the interplay between the respiratory mucosa and GI microbiota is under investigation. Changes in the gut microbiota density and diversity can affect lung immunity, and lung inflammation can lead to intestinal dysbiosis (de Oliveira et al., 2021). However, the precise mechanism by which the infection can disseminate from the gut to the lungs and vice-versa is still unknown.

Regardless, drugs that can act on both the respiratory and GI systems to modulate innate and humoral immunity, inflammation, gut microbiome, and gut barrier would likely be of great assistance in COVID-19. Traditional and evidence-based information shows that aniseed can be used for respiratory problems (e.g., asthma, bronchitis, bronchial secretions, cough, etc.) and digestive complaints (e.g., nausea, indigestion, flatulence, intestinal colic, constipation, etc.). Thus, we speculate that *P. anisum* and its metabolites are interesting drug candidates to address viral infections affecting the gastrointestinal system (Akbar, 2020; Nasır and Yabalak, 2021).

### Prospective use of aniseed on long COVID-19

One study verified that while oropharyngeal SARS-CoV-2 shedding was absent at 4 months, there is evidence of fecal RNA shedding at 4 months (12.7% of participants) and 7 months (3.8%) after the diagnosis. The authors also found that GI symptoms (abdominal pain, nausea, and vomiting) are associated with fecal shedding (Natarajan et al., 2022). The prolonged viral shedding in the GI tract could be involved in the long-term infection and be one of the reasons for the systemic effects of long COVID-19 syndrome. Our current study evaluated 2 weeks of an aniseed formulation on the GI symptoms of COVID-19 patients with positive results. However, we theorize that aniseed formulations could be used for a longer period to protect tissues from damage and prevent viral dissemination from the GI tract to other organs through the gut lining.

### Persian medicine and nutrition

As a holistic school of medicine, PM has a special approach to treating various diseases. In this approach, treatment is started with dietary instructions and lifestyle modifications, with pharmaceutical prescriptions given only if there is no proper response (Tafazoli et al., 2022). Adherence to PM-based treatments requires initial compliance with nutritional instructions and lifestyle guidelines, which are quite difficult to implement considering the pandemic nature of COVID-19 and its psychological burden on the patient and the patient’s family. In this process, the appropriate medicine is selected based on principles such as herbal temperament, disease temperament, etc. Since cold and wet are the predominant temperament in most GI symptoms associated with COVID-19 (e.g., diarrhea or bloating) (Pasalar et al., 2016), the warm and dry temperament cited for anise formulations in PM may aligns with its traditional applications.

Also, considering aniseed as a superfood with beneficial effects like antidiabetic and hypolipidemic ones (Shahrajabian et al., 2019), it may be conceived that long-standing aniseed consumption might ameliorate hazardous coexisting conditions such as diabetes or hyperlipidemia in COVID-19 patients. This may lead to better control of the disease and related GI manifestations.

### Persian medicine and traditional Chinese medicine

There have been numerous clinical studies assessing the effectiveness of botanical drugs from Traditional Chinese Medicine (TCM) in alleviating gastrointestinal (GI) symptoms. For example, a systematic review and meta-analysis focusing on Chinese herbal medicine demonstrated non-beneficial effects in improving GI manifestations such as nausea, vomiting, anorexia, and diarrhea. These results may be attributed to variations in botanical drug metabolites, diverse mechanisms of action, and potential drug-herb interactions across different disciplines (Shi et al., 2021).

## Conclusion

The results of our research revealed that an aniseed powder formulation containing trans-anethole (87%–94%) significantly improved abdominal pain, anorexia, and diarrhea in COVID-19 patients. Given the existing biological properties of *P. anisum* and trans-anethole, we theorize that symptom improvement could have been due to the multitargeted activity and protective mechanism in the GI system. *P. anisum* and its phytochemicals may act directly on the viral infection by inhibiting SARS-CoV-2 S protein and 3CL^PRO^, and could intervene in the COVID-19 pathological process through their anti-inflammatory, antioxidant and hepatoprotective properties, balancing COVID-19-associated dysbiosis, supporting the gut barrier, and promoting a dual therapeutic action in the respiratory and GI systems via the gut-lung axis. Therefore, *P. anisum* could be a safe and effective option for reducing GI symptoms associated with enteric SARS-CoV-2 infection. Larger studies with longer follow-up periods are recommended to confirm these findings and provide additional insights on effects of *P. anisum* on long COVID-19 and its mechanisms of action.

## Data Availability

The raw data supporting the conclusion of this article will be made available by the authors, without undue reservation.

## References

[B1] Aghili Shirazi M. H. (2008).Moalejat-e Aghili [Aghili's Treatments] (Persian) Tehran, Iran: Iran Medical University.

[B2] Aghili Shirazi M. H. (2009). Makhzan-al-advia [Materia Medica] (Persian). Tehran, Iran: Tehran University of Medical Sciences, 801.

[B3] Akbar S. (2020). Handbook of 200 Medicinal Plants. Switzerland: Springer Cham.

[B4] Astani A. Reichling J. Schnitzler P. (2011). Screening for antiviral activities of isolated compounds from essential oils. Evid. Based Complement. Altern. Med. 2011, 253643. 10.1093/ecam/nep187 PMC3096453 20008902

[B5] Avicenna (2005). Al-Qanun fi al-Tibb [The Canon of Medicine] (Persian). Tehran, Iran: Soroush Press.

[B6] Chu C.-C. Hsing C.-H. Shieh J.-P. Chien C.-C. Ho C.-M. Wang J.-J. (2014). The cellular mechanisms of the antiemetic action of dexamethasone and related glucocorticoids against vomiting. Eur. J. Pharmacol. 722, 48–54. 10.1016/j.ejphar.2013.10.008 24184695

[B7] Coates M. D. Lahoti M. Binion D. G. Szigethy E. M. Regueiro M. D. Bielefeldt K. (2013). Abdominal pain in ulcerative colitis. Inflamm. Bowel Dis. 19 (10), 2207–2214. 10.1097/MIB.0b013e31829614c6 23929261 PMC3749243

[B8] Costela-Ruiz V. J. Illescas-Montes R. Puerta-Puerta J. M. Ruiz C. Melguizo-Rodríguez L. (2020). SARS-CoV-2 infection: the role of cytokines in COVID-19 disease. Cytokine Growth Factor Rev. 54, 62–75. 10.1016/j.cytogfr.2020.06.001 32513566 PMC7265853

[B9] Dargahi T. Ilkhani R. Ghiaee A. Arbabtafti R. Fahimi S. Athari S. S. (2021). Anti-inflammatory effect of Pimpinella anisum extract in a mouse model of allergic asthma. Res. J. Pharmacogn. 8 (3), 41–49. 10.22127/RJP.2021.280757.1689

[B10] da Rocha B. A. Ritter A. M. Ames F. Q. Gonçalves O. H. Leimann F. V. Bracht L. (2017). Acetaminophen-induced hepatotoxicity: preventive effect of trans anethole. Biomed. Pharmacother. 86, 213–220. 10.1016/j.biopha.2016.12.014 28006746

[B11] da Rocha E. M. T. Bracht L. Gonçalves O. H. Leimann F. V. Ames F. Q. Schneider L. C. L. (2023). Development and characterization of trans-anethole-containing solid lipid microparticles: antiinflammatory and gastroprotective effects in experimental inflammation. Naunyn Schmiedeb. Arch. Pharmacol. 396 (3), 469–484. 10.1007/s00210-022-02323-2 36385686

[B12] de Oliveira G. L. V. Oliveira C. N. S. Pinzan C. F. de Salis L. V. V. Cardoso C. R. B. (2021). Microbiota modulation of the gut-lung Axis in COVID-19. Front. Immunol. 12, 635471. 10.3389/fimmu.2021.635471 33717181 PMC7945592

[B13] Fang D. Ma J. Guan J. Wang M. Song Y. Tian D. (2020). Manifestations of digestive system of hospitalized patients with coronavirus disease 2019 in Wuhan, China: a single-center descriptive study. Chin. J. Dig. 40 (3), 151–156.

[B14] Gandomkar A. Poustchi H. Moini M. Moghadami M. Imanieh H. Fattahi M. R. (2017). Pars cohort study of non-communicable diseases in Iran: protocol and preliminary results. Int. J. Public Health 62 (3), 397–406. 10.1007/s00038-016-0848-2 27349480

[B15] Ghosh A. Saleh-e-In M. M. Abukawsar M. M. Ahsan M. M. Rahim M. M. Bhuiyan M. N. H. (2019). Characterization of quality and pharmacological assessment of Pimpinella anisum L. (Anise) seeds cultivars. J. Food Meas. Charact. 13, 2672–2685. 10.1007/s11694-019-00188-3

[B16] Ghoshegir S. A. Mazaheri M. Ghannadi A. Feizi A. Babaeian M. Tanhaee M. (2015). Pimpinella anisum in the treatment of functional dyspepsia: a double-blind, randomized clinical trial. J. Res. Med. Sci. 20 (1), 13–21. 25767516 PMC4354059

[B17] Gülçın İ. Oktay M. Kıreçcı E. Küfrevıoǧlu Ö. İ. (2003). Screening of antioxidant and antimicrobial activities of anise (Pimpinella anisum L.) seed extracts. Food Chem. 83 (3), 371–382. 10.1016/s0308-8146(03)00098-0

[B18] Hasan L. K. Deadwiler B. Haratian A. Bolia I. K. Weber A. E. Petrigliano F. A. (2021). Effects of COVID-19 on the musculoskeletal system: clinician’s guide. Orthop. Res. Rev. 13, 141–150. 10.2147/ORR.S321884 34584465 PMC8464590

[B19] Hawrelak J. A. Cattley T. Myers S. P. (2009). Essential oils in the treatment of intestinal dysbiosis: a preliminary *in vitro* study. Altern. Med. Rev. 14 (4), 380–384. 20030464

[B20] Hayashi Y. Wagatsuma K. Nojima M. Yamakawa T. Ichimiya T. Yokoyama Y. (2021). The characteristics of gastrointestinal symptoms in patients with severe COVID-19: a systematic review and meta-analysis. J. Gastroenterol. 56 (5), 409–420. 10.1007/s00535-021-01778-z 33759041 PMC7987120

[B21] Hikmet F. Méar L. Edvinsson A. Micke P. Uhlén M. Lindskog C. (2020). The protein expression profile of ACE2 in human tissues. Mol. Sys Biol. 16 (7), e9610. 10.15252/msb.20209610 PMC7383091 32715618

[B22] Iannarelli R. Marinelli O. Morelli M. B. Santoni G. Amantini C. Nabissi M. (2018). Aniseed (Pimpinella anisum L.) essential oil reduces pro-inflammatory cytokines and stimulates mucus secretion in primary airway bronchial and tracheal epithelial cell lines. Industrial Crops Prod. 114, 81–86. 10.1016/j.indcrop.2018.01.076

[B23] Ibn-al-Nafis A. (2008). Al-Shamel fi Sana'at al-Tibbiyah [Encyclopedia of Materia Medica] (Arabic). Tehran, Iran: Iran Medical University.

[B24] Jamshidzadeh A. Heidari R. Razmjou M. Karimi F. Moein M. R. Farshad O. (2015). An *in vivo* and *in vitro* investigation on hepatoprotective effects of Pimpinella anisum seed essential oil and extracts against carbon tetrachloride-induced toxicity. Iran. J. Basic Med. Sci. 18 (2), 205–211. 25825639 PMC4366734

[B25] Jiao L. Li H. Xu J. Yang M. Ma C. Li J. (2021). The gastrointestinal tract is an alternative route for SARS-CoV-2 infection in a nonhuman primate model. Gastroenterol 160 (5), 1647–1661. 10.1053/j.gastro.2020.12.001 PMC7725054 33307034

[B26] Jin X. Lian J. S. Hu J. H. Gao J. Zheng L. Zhang Y. M. (2020). Epidemiological, clinical and virological characteristics of 74 cases of coronavirus-infected disease 2019 (COVID-19) with gastrointestinal symptoms. Gut 69 (6), 1002–1009. 10.1136/gutjnl-2020-320926 32213556 PMC7133387

[B27] Lee J. B. Yamagishi C. Hayashi K. Hayashi T. (2011). Antiviral and immunostimulating effects of lignin-carbohydrate-protein complexes from Pimpinella anisum. Biosci. Biotechnol. Biochem. 75 (3), 459–465. 10.1271/bbb.100645 21389629

[B28] Luo X. Lv M. Zhang X. Estill J. Yang B. Lei R. (2022). Clinical manifestations of COVID-19: an overview of 102 systematic reviews with evidence mapping. J. Evid. Based Med. 15 (3), 201–215. 10.1111/jebm.12483 35909298 PMC9353366

[B29] Ma C. Cong Y. Zhang H. (2020). COVID-19 and the digestive system. Am. J. Gastroenterol. 115 (7), 1003–1006. 10.14309/ajg.0000000000000691 32618648 PMC7273952

[B30] Ma Q. Xie Y. Wang Z. Lei B. Chen R. Liu B. (2021). Efficacy and safety of ReDuNing injection as a treatment for COVID-19 and its inhibitory effect against SARS-CoV-2. J. Ethnopharmacol. 279, 114367. 10.1016/j.jep.2021.114367 34174375 PMC8223030

[B31] Menon T. Sharma R. Earthineni G. Iftikhar H. Sondhi M. Shams S. (2021). Association of gastrointestinal system with severity and mortality of COVID-19: a systematic review and meta-analysis. Cureus 13 (2), e13317. 10.7759/cureus.13317 33738161 PMC7957843

[B32] Mosaffa-Jahromi M. Bagheri Lankarani K. Pasalar M. Afsharypuor S. Tamaddon A.-M. (2016). Efficacy and safety of enteric coated capsules of anise oil to treat irritable bowel syndrome. J. Ethnopharmacol. 194, 937–946. 10.1016/j.jep.2016.10.083 27815079

[B33] Nasır A. Yabalak E. (2021). Investigation of antioxidant, antibacterial, antiviral, chemical composition, and traditional medicinal properties of the extracts and essential oils of the Pimpinella species from a broad perspective: a review. J. Essent. Oil Res. 33 (5), 411–426. 10.1080/10412905.2021.1928559

[B34] Natarajan A. Zlitni S. Brooks E. F. Vance S. E. Dahlen A. Hedlin H. (2022). Gastrointestinal symptoms and fecal shedding of SARS-CoV-2 RNA suggest prolonged gastrointestinal infection. Med. (New York, NY) 3 (6), 371–387.e9. 10.1016/j.medj.2022.04.001 PMC9005383 35434682

[B35] Pan L. Mu M. Yang P. Sun Y. Wang R. Yan J. (2020). Clinical characteristics of COVID-19 patients with digestive symptoms in hubei, China: a descriptive, cross-sectional, multicenter study. Am. J. Gastroenterol. 115 (5), 766–773. 10.14309/ajg.0000000000000620 32287140 PMC7172492

[B36] Pasalar M. Nimrouzi M. Choopani R. Mosaddegh M. Kamalinejad M. Mohagheghzadeh A. (2016). Functional dyspepsia: a new approach from traditional Persian medicine. Avicenna J. Phytomed 6 (2), 165–174. 27222829 PMC4877961

[B37] Poggiali E. Mateo Ramos P. Bastoni D. Vercelli A. Magnacavallo A. (2020). Abdominal pain: a real challenge in novel COVID-19 infection. Eur. J. Case Rep. Intern Med. 7 (4), 001632. 10.12890/2020_001632 32309266 PMC7162568

[B38] Polonikov A. (2020). Endogenous deficiency of glutathione as the most likely cause of serious manifestations and death in COVID-19 patients. ACS Infect. Dis. 6 (7), 1558–1562. 10.1021/acsinfecdis.0c00288 32463221

[B39] Pontén F. Jirström K. Uhlen M. (2008). The human protein atlas—a tool for pathology. J. Pathol. 216 (4), 387–393. 10.1002/path.2440 18853439

[B40] Rahmanzade R. Rahmanzadeh R. Hashemian S. M. Tabarsi P. (2020). Iran's approach to COVID-19: evolving treatment protocols and ongoing clinical trials. Front. Public Health 8, 551889. 10.3389/fpubh.2020.551889 33014984 PMC7498537

[B41] Samadi-Noshahr Z. Hadjzadeh M. A. Moradi-Marjaneh R. Khajavi-Rad A. (2021). The hepatoprotective effects of fennel seeds extract and trans-Anethole in streptozotocin-induced liver injury in rats. Food Sci. Nutr. 9 (2), 1121–1131. 10.1002/fsn3.2090 33598196 PMC7866591

[B42] Shahrajabian M. H. Sun W. Cheng Q. (2019). Chinese star anise and anise, magic herbs in traditional Chinese medicine and modern pharmaceutical science. Asian J. Med. Biol. Res. 5 (3), 162–179. 10.3329/ajmbr.v5i3.43584

[B43] Shi S. Wang F. Li J. Li Y. Li W. Wu X. (2021). The effect of Chinese herbal medicine on digestive system and liver functions should not be neglected in COVID-19: an updated systematic review and meta-analysis. IUBMB Life 73 (5), 739–760. 10.1002/iub.2467 33725395 PMC8250823

[B44] Sieske L. Janssen G. Babel N. Westhoff T. H. Wirth R. Pourhassan M. (2019). Inflammation, appetite and food intake in older hospitalized patients. Nutrients 11 (9), 1986. 10.3390/nu11091986 31443557 PMC6770921

[B45] Suhail S. Zajac J. Fossum C. Lowater H. McCracken C. Severson N. (2020). Role of oxidative stress on SARS-CoV (sars) and SARS-CoV-2 (COVID-19) infection: a review. Protein J. 39 (6), 644–656. 10.1007/s10930-020-09935-8 33106987 PMC7587547

[B46] Sun Z. Song Z. G. Liu C. Tan S. Lin S. Zhu J. (2022). Gut microbiome alterations and gut barrier dysfunction are associated with host immune homeostasis in COVID-19 patients. BMC Med. 20 (1), 24. 10.1186/s12916-021-02212-0 35045853 PMC8769945

[B47] Suzuki Y. Saito E. Wakabayashi T. Suwa A. (2007). Prevention and treatment of NSAIDs-induced gastrointestinal complications by prostaglandin derivatives. Nihon Rinsho 65 (10), 1843–1849. 17926534

[B48] Sweetser S. (2012). Evaluating the patient with diarrhea: a case-based approach. Mayo Clin. Proc. 87 (6), 596–602. 10.1016/j.mayocp.2012.02.015 22677080 PMC3538472

[B49] Tafazoli V. Tavakoli A. Mosaffa-Jahromi M. Cooley K. Pasalar M. (2022). Approach of Persian medicine to health and disease. Adv. Integr. Med. 9 (1), 3–8. 10.1016/j.aimed.2021.07.007

[B50] Tas A. Ozbek H. Atasoy N. Altug M. E. Ceylan E. (2006). Evaluation of analgesic and anti inflammatory activity of Pimpinella anisum fixed oil extract. Indian Vet. J. 83 (8), 840–843.

[B51] Tong Y. Yu C. Chen S. Zhang X. Yang Z. Wang T. (2023). Trans-anethole exerts protective effects on lipopolysaccharide-induced acute jejunal inflammation of broilers via repressing NF-κB signaling pathway. Poult. Sci. 102 (2), 102397. 10.1016/j.psj.2022.102397 36565631 PMC9801195

[B52] Wang Y. Li Y. Zhang Y. Liu Y. Liu Y. (2022). Are gastrointestinal symptoms associated with higher risk of Mortality in COVID-19 patients? A systematic review and meta-analysis. BMC Gastroenterol. 22 (1), 106. 10.1186/s12876-022-02132-0 35255816 PMC8899790

[B53] WHO (2021). COVID-19 clinical management: living guidance, 25 January 2021. Geneva: World Health Organization.

[B54] Yang C. Cai L. Xiao S. Y. (2023). Pathologic characteristics of digestive tract and liver in patients with coronavirus disease 2019. Gastroenterol. Clin. North Am. 52 (1), 201–214. 10.1016/j.gtc.2022.09.003 36813426 PMC9531645

[B55] Yi Q. Liu J. Zhang Y. Qiao H. Chen F. Zhang S. (2021). Anethole attenuates enterotoxigenic Escherichia coli-induced intestinal barrier disruption and intestinal inflammation via modification of TLR signaling and intestinal microbiota. Front. Microbiol. 12, 647242. 10.3389/fmicb.2021.647242 33841372 PMC8027122

[B56] Yu C. Wang D. Li Q. Tong Y. Yang Z. Wang T. (2022). Trans-anethole ameliorates LPS-induced inflammation via suppression of TLR4/NF-κB pathway in IEC-6 cells. Int. Immunopharmacol. 108, 108872. 10.1016/j.intimp.2022.108872 35617845

[B57] Zeng W. Qi K. Ye M. Zheng L. Liu X. Hu S. (2022). Gastrointestinal symptoms are associated with severity of coronavirus disease 2019: a systematic review and meta-analysis. Eur. J. Gastroenterol. Hepatol. 34 (2), 168–176. 10.1097/MEG.0000000000002072 33470700

[B58] Zhong P. Xu J. Yang D. Shen Y. Wang L. Feng Y. (2020). COVID-19-associated gastrointestinal and liver injury: clinical features and potential mechanisms. Signal Transduct. Target Ther. 5 (1), 256. 10.1038/s41392-020-00373-7 33139693 PMC7605138

[B59] Zuo T. Zhang F. Lui G. C. Y. Yeoh Y. K. Li A. Y. L. Zhan H. (2020). Alterations in gut microbiota of patients with COVID-19 during time of hospitalization. Gastroenterol 159 (3), 944–955. 10.1053/j.gastro.2020.05.048 PMC7237927 32442562

